# Home-based (virtual) rehabilitation improves motor and cognitive function for stroke patients: a randomized controlled trial of the *Elements* (EDNA-22) system

**DOI:** 10.1186/s12984-021-00956-7

**Published:** 2021-11-25

**Authors:** Peter H. Wilson, Jeffrey M. Rogers, Karin Vogel, Bert Steenbergen, Thomas B. McGuckian, Jonathan Duckworth

**Affiliations:** 1 Healthy Brain and Mind Research Centre (HBMRC) and School of Behavioural and Health Sciences, Australian Catholic University, Melbourne, VIC Australia; 2 Faculty of Health Sciences, The University of Sydney, Sydney, NSW Australia; 3 Prince of Wales Hospital, Sydney, NSW Australia; 4 Behavioural Science Institute, Radboud University, Nijmegen, The Netherlands; 5 School of Design, RMIT, Melbourne, VIC Australia

**Keywords:** Cognition, Motor activity, Rehabilitation, Stroke, Upper extremity, Virtual rehabilitation

## Abstract

**Background:**

Home-based rehabilitation of arm function is a significant gap in service provision for adult stroke. The EDNA-22 tablet is a portable virtual rehabilitation-based system that provides a viable option for home-based rehabilitation using a suite of tailored movement tasks, and performance monitoring via cloud computing data storage. The study reported here aimed to compare use of the EDNA system with an active control (*Graded Repetitive Arm Supplementary Program*—GRASP training) group using a parallel RCT design.

**Methods:**

Of 19 originally randomized, 17 acute-care patients with upper-extremity dysfunction following unilateral stroke completed training in either the treatment (n = 10) or active control groups (n = 7), each receiving 8-weeks of in-home training involving 30-min sessions scheduled 3–4 times weekly. Performance was assessed across motor, cognitive and functional behaviour in the home. Primary motor measures, collected by a blinded assessor, were the Box and Blocks Task (BBT) and 9-Hole Pegboard Test (9HPT), and for cognition the Montreal Cognitive Assessment (MoCA). Functional behaviour was assessed using the Stroke Impact Scale (SIS) and Neurobehavioural Functioning Inventory (NFI).

**Results:**

One participant from each group withdrew for personal reasons. No adverse events were reported. Results showed a significant and large improvement in performance on the BBT for the more-affected hand in the EDNA training group, only (*g* = 0.90). There was a mild-to-moderate effect of training on the 9HPT for EDNA (*g* = 0.55) and control (*g* = 0.42) groups, again for the more affected hand. In relation to cognition, performance on the MoCA improved for the EDNA group (*g* = 0.70). Finally, the EDNA group showed moderate (but non-significant) improvement in functional behaviour on the SIS (*g* = 0.57) and NFI (*g* = 0.49).

**Conclusion:**

A short course of home-based training using the EDNA-22 system can yield significant gains in motor and cognitive performance, over and above an active control training that also targets upper-limb function. Intriguingly, these changes in performance were corroborated only tentatively in the reports of caregivers. We suggest that future research consider how the implementation of home-based rehabilitation technology can be optimized. We contend that self-administered digitally-enhanced training needs to become part of the health literacy of all stakeholders who are impacted by stroke and other acquired brain injuries.

*Trial registration* Australian New Zealand Clinical Trials Registry (ANZCTR) Number: ACTRN12619001557123. Registered 12 November 2019, http://www.anzctr.org.au/Trial/Registration/TrialReview.aspx?id=378298&isReview=true

## Background

Stroke survivors regard the rehabilitation of upper-limb function as one of the top priorities for increasing their quality of life [1]. However, during the rehabilitation phase the time spent engaged in functional upper-limb activities is often low [2, 3], and at six months after stroke up to 70% remain unable to regain functional use of their affected upper limb(s) [4, 5]. Barriers to therapy include limited access to services, particularly after the transition from acute to in-home care, and low levels of engagement in the rehabilitation program/task itself. These issues have been further compounded by the COVID-19 pandemic, which has highlighted the lack of interventions capable of simultaneously engaging patients in therapy while affording the social distancing and domiciliary options essential for continuity of health care.

Current research shows that optimal recovery from an *acquired brain injury* (ABI) can be achieved when tailored rehabilitation is provided at high intensity and over a sustained period [7, 8]. Moreover, training tasks should be scaled in complexity (both motor and cognitive) in a manner that accords with the individual needs and capabilities of the patient, fostering motivation and continued progression. To this end, tailored *virtual reality* (VR), *augmented reality* (AR) and associated interactive technology can provide a number of key assets for rehabilitation, most notably a medium to increase training doses during critical phases of recovery, scale task difficulty in a systematic way, engage patients’ interest in novel forms of interaction, enhance learning via use of augmented feedback, and record the progress of patients using system-generated metrics. A design principle for many such systems is the notion of *enriched therapeutic environments* to promote skill acquisition and transfer [9]. The notion here is to present a task environment that not only affords physical movement but also engages the patient’s cognitive attention—both are critical ingredients in skilled performance. This is supported by a recent systematic review and meta-analysis that showed enhanced motor outcomes when these critical ingredients are met with purpose-designed systems [8].

The *Elements* system (aka EDNA™) [10] was designed originally as a tabletop device (using tangible interfaces) for clinic-based rehabilitation of ABI, with earlier evaluations showing its efficacy for *traumatic brain injury* [11, 12] and adult stroke [13]. Targeting upper-limb function in TBI patients, significant gains in motor skill were demonstrated in case study [11] and within-groups evaluations [12]. Gains in upper-limb skill also showed positive transfer to everyday function. The most recent RCT extended the application of EDNA to adult stroke and showed strong treatment effects across motor, cognitive and functional outcomes [13]. As well, the experience of using EDNA has been rated highly on the Virtual User Experience Questionnaire (VUE-Q), adapted from the Presence Scale of Witmer and colleagues [14, 15]. Patients have rated highly all six sub-scales: Familiarity, Enjoyment/Engagement, Controllability/Affordance, Efficacy, Social Engagement, and Immersion/Presence, suggesting the system is able to effectively engage the user in the rehabilitation program and promote a sense of improvement. Among the limitations of this in-clinic application, however, is the requirement for one-to-one administration of an adjunct treatment, placing additional demands on the time and resources of rehabilitation services [16].

The capacity to extend access to training into the home environment is particularly important for stroke patients who routinely fail to achieve the recommended doses or durations of therapy necessary to promote meaningful gains [2, 3]. So-called *telerehabilitation* systems encompass a variety of modalities from videoconferencing, health literacy training delivered over the web, and VR-based systems. Research to date on the benefits of VR- and AR-based therapy in the home are encouraging, but very few controlled trials exist [17]. Evidence suggests, however, that the benefits of such treatment for motor and cognitive function are at least equivalent to standard physical therapy or home-based exercise. In terms of implementation, some guiding principles include the need to “design for engagement” and accommodating the practical challenges of use in the home [18]. These principles speak to the portability of the device and ease of use, providing a viable option for continuity of care as patients transition from the hospital to the home [19].

The EDNA system has therefore recently been extended to include a transportable, tablet device (EDNA-22) for home-based delivery. Targeting upper-limb impairments, the system is designed to provide a viable and flexible therapy option in multiple environments (e.g., clinic, home, and community), which is critical if patients are to achieve recommended doses and durations of rehabilitation [20]. A customised and regular schedule of therapy is delivered via the internet to the patient in their residence, performance data is collected and stored in the cloud, and adherence and performance data is relayed back to the therapist in the clinic. The broad aim of the study presented here was to evaluate the motor, cognitive and functional outcomes of an intensive course of home-based rehabilitation using the EDNA-22 system. First, based on our earlier clinical trials, we expected that participants recovering from stroke would be able to engage effectively in the home-based therapy and adhere to the complete course (i.e., minimum 3 sessions per week, recorded with written log). Second, we expected that the course of therapy would produce significant gains in motor and cognitive function, measured using standardized and validated clinical tools, with the magnitude of changes greater than that observed for an active control therapy (*Graded Repetitive Arm Supplementary Program*—GRASP) [21, 22]. Third, we expected that patients would also report positive changes in their level of motor functioning. Fourth, we expected that caregivers would report positive change in the general everyday function of patients as a result of the therapy. Finally, we expected the training benefits of EDNA to be maintained across motor, cognitive and functional outcomes at a short-term follow-up (3 months).

## Methods

This study used a parallel RCT design, comparing the use of the EDNA take-home system with an active control GRASP training group. The study was approved by the relevant hospital (HREC 18/241) and university Human Research Ethics Committees, and performed in accordance with their guidelines. Prior to commencing, the trial was registered with the Australian New Zealand Clinical Trials Registry (Project Record 378298).

### Participants

**Fig. 1:**
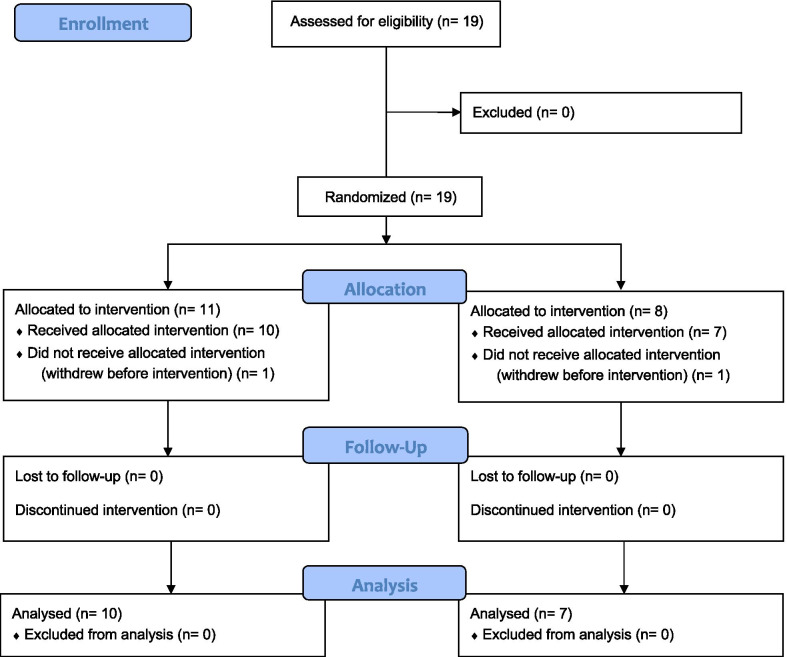
CONSORT participant flow diagram

Nineteen acute-care stroke patients were initially recruited for the study between November 2019 and February 2021; nine started as inpatients of a large tertiary hospital in Sydney (Prince of Wales Hospital), and 10 as outpatients (one of whom referred by a community health program). Participant recruitment was ended pragmatically in view of the prospect of continued disruptions from the COVID-19 pandemic in Australia. Inclusion criteria were: (1) upper-extremity dysfunction following a unilateral stroke, confirmed by neuroimaging, and expressed a specific goal to address these deficits in rehabilitation; (2) spoken English as either a first or second language, and the ability to understand and follow oral instructions; (3) ability to maintain sitting balance without assistance, and (4) a minimum range of upper-limb movement including 20° of active shoulder flexion, ability to maintain elbow flexion at 90°, and ability to maintain the wrist in a neutral position while holding an object used by the EDNA system, assessed by the study occupational therapist (KV) prior to randomization. The ability to form a mass grasp was a desirable criterion, but not essential as an adjustable strap (attached to the object) was available for use when grasping was difficult. Exclusion criteria were: (1) a prior neurological disorder (other than stroke), psychiatric or developmental disorder; (2) disturbances in visual function that would prevent task completion; or (3) less than 18 years of age. Rehabilitation staff referred eligible patients into the study. All participants were living at their residence (either home or nursing home). All participants (or their caregivers) provided written informed consent prior to their participation. Upon entry into the study, each patient was allocated randomly to either a treatment group (receiving the take-home EDNA training) or an active control group (receiving the GRASP training); see Procedure for details. The EDNA treatment group comprised of 11 patients who completed all pre-test assessments; of these, one withdrew for personal reasons prior to the first treatment session, and 10 completed the take-home training and post-test assessment. The GRASP control group comprised 8 patients who completed pre-test assessment; of these, one patient withdrew for personal reasons. All participants that started at-home training adhered to a minimum of three sessions and maximum of four per week. Participant flow is outlined in Fig. 1.

### Participant characteristics

The *NIH Stroke Scale* (NIHSS) was used to quantify stroke severity [23]. This is an 11-item scale that assesses level of consciousness, and visual, motor, sensory and language function. Higher scores (max = 42) indicate more severe symptoms. The NIHSS was completed by the treating team at hospital admission.

*Functional independence measure (FIM)* is an 18-item assessment of the amount of assistance an individual requires to perform activities of daily living. Validated for use in stroke [24], lower FIM scores reflect greater levels of disability. FIM was completed by the treating team at the commencement of the rehabilitation episode.

*ABILHAND* is a patient-reported questionnaire that assesses the individual patient’s perceived difficulty in performing everyday bimanual activities without assistance [25]. The scale presents 23 bimanual activities and asks the patient to rate the ease of performance for each item on a 3-point scale (impossible = 0, difficult = 1, easy = 2). Across the 23-items, higher scores (max = 46) reflect better manual ability.

*The Fatigue Severity Scale* (FSS) is a 9-item Likert-based instrument (1 = strongly disagree to 7 = strongly agree) examining the impact and severity of fatigue, as reported by the patient [26]. Higher scores reflect greater subjective sense of tiredness and lack of energy.

### Outcome measures

#### Motor performance

The *Box and block test (BBT)* is a well-validated test of (unimanual) upper-limb function in both clinical and non-clinical groups. The performer is required to move manually as many 2.5-cm wooden cubes from one box to another with one hand in 60sec. The BBT has exceptional test–retest reliability (*r* = 0.96), and inter-rater reliability (*r* = 0.99) [27, 28]. Importantly, scores on the BBT correlate very highly with total scores on standardised tests of upper-limb function: Spearman’s rho of 0.95 with the Action Research Arm Test, and 0.92 with the Fugl-Meyer motor scale [28]. Higher scores on the BBT reflect better upper-limb motor function. Minimal Detectible Change (MDC) for stroke patients is 5.5 blocks/minute for the more affected hand, and 7.8 blocks/minute for the less affected hand [29].

The *9-Hole Peg Test* (9-HPT) is a widely used clinical measure of fine-motor (or manual) control. Participants are required to grasp pegs from a container and place them one by one into holes on a board, as quickly as possible. Participants must then remove the pegs from the holes, one by one, and replace them back into the container. Results were expressed as the number of pegs placed per second (peg/s) with higher scores (18 pegs/total time) reflecting greater fine manual control [30]. For stroke patients, MDC is 0.27 pegs/s for the more affected hand, and 1.45 pegs/s for the less affected hand [29].

#### Cognitive performance

The *Montreal Cognitive Assessment* (MoCA) [31] is a brief screening tool that provides a general measure of intellectual function. The MoCA has shown excellent sensitivity (90%) and specificity (87%) in detecting cognitive impairment [32] and monitoring recovery following stroke [33].

#### Functional performance

The *Stroke Impact Scale* (SIS) provides a self-report measure of health-related quality of life (HRQoL) [34]. The SIS includes a visual analogue scale to determine the extent to which participants feel they have recovered from their stroke (range 0–100). Higher scores represent a greater sense of global recovery.

*Neurobehavioural function inventory* (NFI) [35] is a 76-item measure of functional behaviour and symptoms in everyday life that are commonly encountered after brain injury. The NFI has six sub-scales (Depression; Somatic; Memory/Attention; Communication; Aggression; Motor), and is also frequently used as a general measure of functional adaptation. In our study, the Total Score of the informant report version of the NFI was used to measure the frequency of difficulties perceived by a family member.

### Training procedure

#### Randomization and data acquisition

For this parallel RCT, patients were stratified by age and type of stroke (ischemic or hemorrhagic), and then randomly allocated to the treatment (EDNA + Treatment As Usual—TAU) or control (GRASP + TAU) group [21, 22]. Concealed block randomization (1:1) was completed by breaking sequentially-numbered sealed envelopes, prepared by the study coordinator (JMR) using a random number generator (https://www.sealedenvelope.com/simple-randomiser/v1/lists). Outcome measures were collected by an assessor blinded to treatment allocation. Using medical chart review and patient interview, participant characteristic information on sociodemographic and medical history, neurological and radiological data were collected. Assessment of motor, cognitive, and functional outcomes occurred at three time points: prior to in-home training (pre-test); immediately following training (post-test); and, three months after the completion of training (follow-up). Pre-test data were collected in hospital, while post-test and follow-up data were collected in home settings.

#### EDNA training in the home

**Fig. 2:**
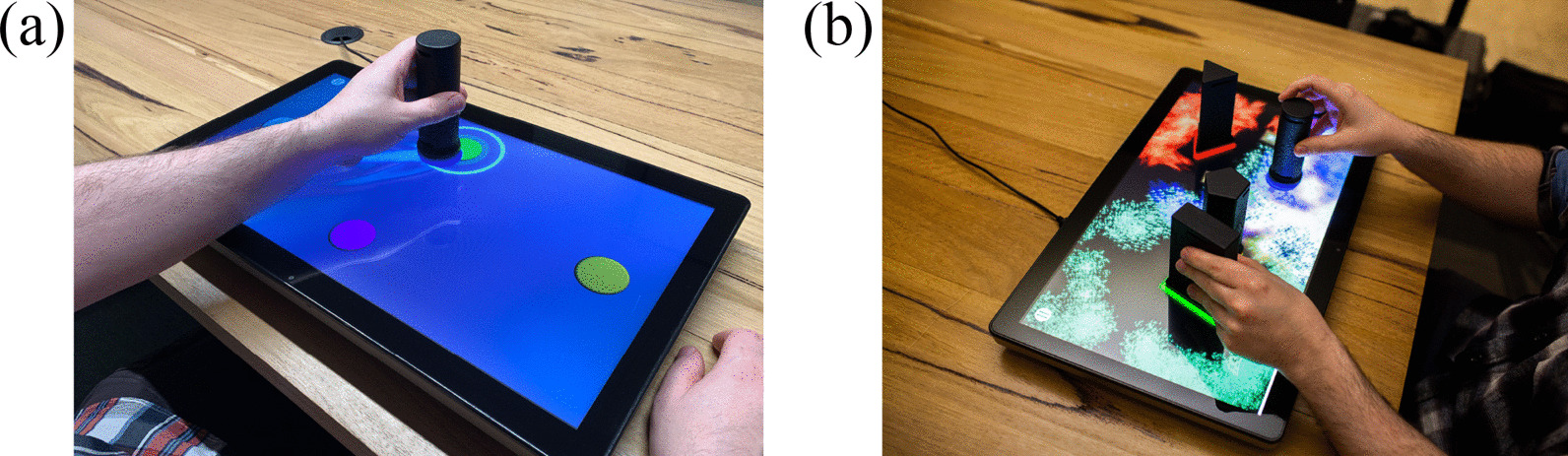
The EDNA-22 system showing **a** goal-directed Random Bases task with visual augmented feedback, and **b** exploratory Swarm task

EDNA training consisted of 30-min of upper-limb training per session, with a minimum of three and maximum of four sessions per week, for an 8-week period. All patients allocated to EDNA received either an initial set-up and training session at the hospital before discharge, or in their home. This training addressed any questions regarding the physical location of the system or connectivity issues. There was weekly phone contact between the research team occupational therapist (KV, not blinded to group allocation) and the patient (and/or caregiver) in the home to address any questions about the set-up or operation of the system. The display technology for presentation of task environments consists of a 22-inch touchscreen tablet (Elo™ I-series) (Fig. 2). The EDNA software is programmed using Unity™ operating on the MS Windows 10 platform, with secure cloud-based data collection (Azure™, MS Inc.).

The EDNA training tasks consist of four goal-based and three exploratory movement activities that require manipulation of handheld objects (or *tangible user interfaces*—TUIs) on the surface of the display; the display itself is placed horizontally on a table in front of the seated patient. Unimanual movements involving the more-affected and less-affected hand are required, together with bimanual movement for the exploratory tasks. The four goal-directed tasks are as follows: Task 1 (Bases) consisted of the home base and four potential movement targets, all 78 mm in diameter. The circular targets were cued in a fixed order (east, north, west, south) using an illuminated border. Task 2 (Random Bases) had the same configuration of targets, but they were highlighted in random order. Task 3 (Chase Task) began with a blank screen. A target circle then appeared randomly in one of nine locations. These locations were configured along three radials emanating from the home base. Task 4 (Go-NoGo) used the same target positions as Task 3, however, additional distractor targets (a pentagon, triangle and rectangle) appeared. Participants were instructed to place the object on the circular targets only and to resist moving to distractors [13]. Tasks 5, 6 and 7 require participants to explore the virtual environment creatively to make novel visual and auditory effects through movement and placement of the objects [11, 36]. The exploratory environments place greater demands on the volition of the participant by relaxing both goal constraints and environmental prompts, while providing the motivational incentive to create one’s own audiovisual feedback effects through playful movement [12]. Task 5 (Mixer) consists of nine circular targets arranged in a 3 × 3 pattern. The participant places the object on a target to activate a musical sound. The pitch and volume of the sound can be adjusted according to the object’s proximity to the center of the target. Participants can activate and deactivate different combinations of targets by placing, lifting and sliding the object to produce more complex audio effects. Task 6 (Paint) encourages participants to paint and draw using a combination of objects. Each object, when moved across the display, draws unique animated patterns, lines and sound. Task 7 (Swarm) encourages bimanual control to explore the audiovisual relationship between the objects. When an object is placed on the display, multiple coloured shapes slowly gravitate (or swarm) around its base (see Fig. 2b). As each object is moved, its own swarm follows. The movement, colour, size and sound characteristics of each swarm changes as the distance between objects is altered.

#### GRASP control group training in the home

Control group participants participated in 30-min sessions of a GRASP [37] program over an 8-week period (at a schedule of 3 to 4 sessions per week). Hence, total treatment time was matched to that of the EDNA training regime. GRASP is an arm and hand exercise program for people who have suffered a stroke, undertaken by the patient on their own, as an adjunct to their conventional rehabilitation therapies. Independent RCT analyses have demonstrated the GRASP program to benefit the motor recovery of people with sub-acute stroke [21]. Participants in the GRASP group all received an initial set-up and training session to familiarise them with materials, written exercises, and recording sheet. During the training, the study occupational therapist (KV, not blinded to group allocation) maintained weekly telephone contact with each patient to encourage compliance and to troubleshoot any difficulties. Upper limb improvement was assessed periodically with clinical observation, and GRASP levels were changed if clinical criteria were met.

### Data analysis

In light of results from earlier studies of VR-based rehabilitation, we expected a large treatment effect (Cohen’s *d* > 0.80) on primary measures of motor function including the Box and Block test [38–40]. On this basis, and with a desired power of 0.80, we determined that a sample size of 10–12 participants per group was adequate (G*Power 3.1.7) [41]. This calculation was also consistent with the scale of recent proof-of-concept studies of virtual rehabilitation for stroke [42–44].

All data was checked for and satisfied normality assumptions according to Shapiro–Wilk’s and Levene’s tests. The analysis was conducted in three parts. First, pre-test demographic, neurological and functional characteristics of patients were presented descriptively (Table 1) and compared between groups using a series of parametric (*t*-tests) and non-parametric (Chi Square) tests. Second, within each group (EDNA and GRASP), the significance of pre-test to post-test and post-test to follow-up change on each primary outcome measure was analyzed using dependent *t*-tests, and the magnitude of each effect reported as Hedges’ *g* (Table 2). Third, for between-group comparison, individual *change scores* on primary motor, cognitive and functional outcomes were first calculated as the difference between pre-test and post-test performance; mean change scores were then compared between groups using independent-samples *t*-tests. To control the rate of false positives in the planned multiple comparisons, the Benjamini–Hochberg procedure [13, 45], with a false discovery rate of 0.07, was applied to determine the number of significant results. To temper the interpretation of significance tests, estimates of effect size (*g*) were calculated and interpreted according to the conventions of Cohen [46]: *small* ≥ 0.2; *medium* ≥ 0.5; and, *large* ≥ 0.8.

**Table 1:** Demographic, neurological, and functional characteristics of the treatment and control groups at pre-test

	EDNA treatment (*n* = 10)	GRASP control (*n* = 7)	Comparison test
Age (years)^a^	69.9 (13.8), 49–90	77.3 (8.9), 60–85	*t* = 1.241, *p* = 0.234
Gender^b^			*χ*^2^ = 0.004, *p* = 0.949
Male	7	5	
Female	3	2	
Rehab NIHSS^a^	6.7 (3.0)	7.8 (5.0)	*t* = 0.494, *p* = 0.631
Admission FIM score^a^	45.9 (24.8)	40.6 (18.7)	*t* = 0.480, *p* = 0.638
Abilhand	21.1 (10.5)	36.4 (10.4)	*t* = 2.472, *p* = 0.051
FSS Total	38.7 (13.5)	30.3 (2.2)	*t* = 1.212, *p* = 0.249
Time since stroke (days)^a^	137.5 (152.4)	107.4 (56.4)	*t* = 0.496, *p* = 0.627
Stroke type^b^			*χ*^2^ = 0.977, *p* = 0.323
Ischemic stroke^b^	9 (90%)	5 (71%)	
Hemorrhagic stroke^b^	1	2	
Side of lesion: left vs. right	6 left: 4 right	5 left: 2 right	*χ*^2^ = 0.235, *p* = 0.627
BBT, MAH^a^	23.5 (9.6)	15.7 (10.8)	*t* = 1.568, *p* = 0.138
BBT, LAH^a^	41.2 (14.4)	36.6 (10.8)	*t* = 0.720, *p* = 0.482
9HPT, MAH^a^	0.21 (0.20)	0.08 (0.14)	*t* = 1.462, *p* = 0.164
9HPT, LAH^a^	0.58 (0.27)	0.48 (0.15)	*t* = 0.857, *p* = 0.405
MoCA^a^	18.5 (5.1)	17.0 (7.8)	*t* = 0.491, *p* = 0.630
SIS Recovery Rating	55.5 (16.4)	45.0 (20.7)	*t* = 1.125, *p* = 0.279
NFI Total^c^	155.6 (38.1)		

*NIHSS* National Institute of Health Stroke Scale range 0–24; *FIM* Functional Independence Measure; *FSS* Fatigue Severity Scale; *BBT* Box and Block Test; *MAH* More Affected Hand; *LAH* Less Affected Hand; *9HPT* Nine Hole Peg Test (pegs/s); *MoCA* Montreal Cognitive Assessment; *SIS* Stroke Impact Scale; *NFI* Neurobehavioral Functioning Inventory

^a^Mean (SD), Range

^b^No. (%)

^c^NFI data not available for GRASP group

**Table 2:** Primary motor, cognitive and functional outcomes for the EDNA treatment and GRASP control groups at pre-test, post-test and follow-up

Primary outcome^a^	EDNA treatment (*n* = 10)					GRASP control (*n* = 7)					Group effect on pre-post change score (*t*, *p*)^c^
	Pre-test	Post-test	Follow-up	Pre-post Change score^b^	Effect size^d^	Pre-test	Post-test	Follow-up	Pre-post change score^b^	Effect size^d^	
Motor											
BBT-MAH	23.5 (9.6)	34.7 (13.8)	35.5 (12.8)	11.2 (9.8) *p* = 0.006*	0.90	15.7 (10.8)	16.0 (9.4)	17.9 (8.1)	− 0.3 (9.9) *p* = 0.942	0.03	*t* = 2.25, *p* = 0.040*
BBT-LAH	41.2 (14.4)	46.9 (10.9)	48.8 (12.5)	5.7 (13.2) *p* = 0.205	0.43	36.6 (10.8)	36.0 (12.5)	38.4 (9.7)	0.6 (7.8) *p* = 0.853	0.05	*t* = 1.12, *p* = 0.280
9HPT-MAH	0.21 (0.20)	0.34 (0.25)		0.13 (0.20) *p* = 0.072	0.55	0.08 (0.14)	0.14 (0.13)		0.06 (0.11) *p* = 0.219	0.42	*t* = 0.97, *p* = 0.348
9HPT-LAH	0.58 (0.27)	0.59 (0.25)		0.01 (0.24) *p* = 0.989	0.04	0.48 (0.15)	0.49 (0.08)		0.01 (0.01) *p* = 0.840	0.08	*t* = -0.10, *p* = 0.922
Cognitive											
MoCA	18.5 (5.1)	21.9 (4.1)	23.0 (5.0)	3.4 (3.0) *p* = .006*	0.70	17.0 (7.8)	18.7 (6.0)	21.1 (5.6)	0.1 (3.2) *p* = 0.911	0.23	*t* = 2.31, *p* = 0.036*
Functional											
SIS—Recovery	55.5 (16.4)	64.8 (15.0)		9.3 (14.3) *p* = 0.069	.57	45.0 (20.7)	43.8 (21.3)		− 1.2 (1.9) *p* = 0.201	0.05	*t* = 2.291, *p* = 0.046
NFI—Informant (Total)	155.6 (38.1)	138.0 (29.8)	136.1 (37.3)	− 17.6 (19.5) *p* = 0.107	.49						

*BBT* Box and Block Test; *LAH* Less Affected Hand; *MAH* More Affected Hand; *9HPT* Nine Hole Peg Test (pegs/s); *MoCA* Montreal Cognitive Assessment; *SIS* Stroke Impact Scale; *NFI* Neurobehavioral Functioning Inventory

^a^Mean (*SD*)

^b^Dependent-samples *t*-test comparing pre-test vs. post-test

^c^Independent-samples *t*-test comparing EDNA vs. GRASP

^d^Hedges’ *g*

*p < Benjamini–Hochberg critical value

## Results

### Demographic, neurological, and functional characteristics of the treatment and control groups at pre-test

Full patient characteristics at pre-test are presented in Table 1. The demographic and neurological characteristics of the patients were comparable between the EDNA treatment and GRASP Control groups. The motor and cognitive performance of patients at pre-test was also comparable between groups; the one exception was a non-significant trend shown on the Abilhand (*p* = 0.051). The level of neurobehavioural impairment reported by caregivers on the NFI was high for the EDNA treatment group; only four respondents completed the NFI for the Control group and, as such, the mean score is not presented. No adverse events were reported by participants in either group.

### Primary motor, cognitive and functional outcomes for the EDNA treatment and GRASP control groups at pre-test, post-test and follow-up

**Fig. 3:**
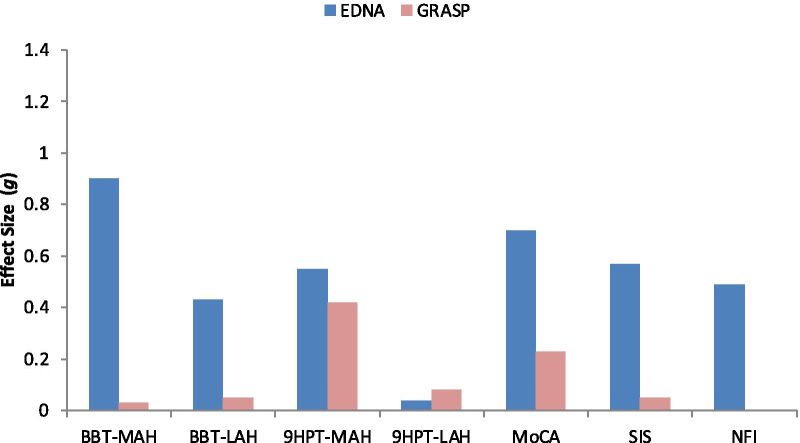
Effect size estimates (Hedges’ *g*) for pre-post change on measures of motor, cognitive and functional performance. (*Note* NFI data was not available for the GRASP group)

There was no significant difference in the total number of training sessions completed by patients in the EDNA (28.0) and GRASP (25.6) groups, *t* < 1. Performance outcomes for the EDNA and GRASP groups at pre-test, post-test and follow-up are presented in Table 2. Effect size estimates for pre-post change on measures of motor, cognitive and functional performance are presented in Fig. 3.

### Pre-post differences

#### Motor outcomes

On the BBT, the EDNA group showed significant pre-post improvement for the more affected hand (MAH), with a high effect size (*g* = 0.90); comparatively, change for the less affected hand (LAH) was mild (*g* = 0.43) and non-significant. Seven out of 10 (MAH) and 4/10 (LAH) of participants improved by more than the MDC. The GRASP group showed negligible (and non-significant) change for each hand, with 3/7 (MAH) and 1/7 (LAH) improving more than the MDC.

On the 9HPT, the level of improvement for the MAH was moderate in the EDNA group (*g* = 0.55) and approached significance (*p* = 0.072), with 5/10 improving more than the MDC. The corresponding effect was mild (*g* = 0.42) and non-significant for the GRASP group, with 1/7 improving more than the MDC. For the LAH, there was negligible (and non-significant) change in 9HPT performance for each group, and zero participants improved more than the MDC.

#### Cognitive outcomes

For the MoCA, significant improvement was evident for the EDNA group, only; the magnitude of this effect was moderate to large (*g* = 0.70).

#### Functional behaviour

SIS results showed a moderate (*g* = 0.57, *p* = 0.069) improvement in HRQoL for the EDNA group, while the GRASP group showed negligible change. NFI data showed that caregivers of patients in the EDNA group reported a moderate (*g* = 0.49) (but non-significant) improvement in functional behaviour in the home.

### Post-test vs. follow-up

For each training group, follow-up data was available for the BBT and MoCA. Within each group, paired-samples *t*-tests were computed to examine whether training outcomes were maintained over the interval between post-test and follow-up. All tests revealed no significant changes in motor/cognitive performance or functional behaviour (each *p* > 0.10). The lack of significant change represents maintenance of gains. The one exception was for the GRASP group on the MoCA, who unexpectedly did not improve over the training phase, but did improve from post-test to follow-up (*p* = 0.012), or after training ceased.

## Discussion

The study reported here sought to evaluate the efficacy of a novel take-home system (EDNA-22) for rehabilitation of adult stroke. Using a RCT design, we evaluated a short course of EDNA training delivered over an 8-week period against an active control group using GRASP training. Results were broadly consistent with our starting hypotheses. First, all patients in the EDNA group were able to complete the course of training successfully in the home. Second, EDNA training conferred significant (pre-post) gains in motor and cognitive performance; notably, a large effect size was observed for the more affected hand on our primary motor measure (BBT: *g* = 0.90), a medium effect on our primary cognitive measure (MoCA: *g* = 0.70), and a non-significant trend on our secondary motor measure (9HPT: *g* = 0.55). By comparison, the GRASP group showed no significant changes in motor or cognitive function, notwithstanding a mild-to-moderate effect size on the 9HPT for the more affected hand. Third, patients in the EDNA group reported positive changes in their level of motor functioning. Fourth, caregiver reports on the NFI were not conclusive but did show a moderate (non-significant) change in general behavioural function as a result of the therapy. Finally, training-related changes for the EDNA group were maintained over a (short-term) 3-month follow-up period. In the discussion that follows, we focus on the motor and cognitive benefits of EDNA training, and compare the nature and magnitude of these changes against similar approaches to take-home rehabilitation.

The primary changes in motor function (assessed on the BBT and 9HPT) accord well with effects that have been observed using similar VR-augmented rehabilitation systems in the home. Piron and colleagues [47], for example, showed a moderate effect size (*d* = 0.62) on the Fugl-Meyer test for a combined VR and tele-rehab approach for treatment of stroke patients with a paretic arm. The treatment in this case spanned four weeks but comprised 5 × 60-min training sessions per week. Of those studies reviewed by Aminov [8], only Standen [48] was conducted in the home. This study evaluated a customized game-based system comprising a virtual-glove and Wiimote tracking system (Nintendo™). The intervention itself was intensive: daily training (up to 60 min), over an 8-week period. Results on the Wolf motor test were encouraging, but significant only for grip strength when compared with usual care. Unfortunately, it was not possible to calculate within-treatment effect sizes for the Standen study. Similarly, using customized videogames and video-capture technology, Kizony and colleagues [49] reported significant within-group changes on the Fugl–Meyer upper extremity sub-test and functional use of the upper-limb in everyday activity supporting positive transfer. In short, related interventions using VR show comparable gains in motor performance to those reported in our study.

The cognitive benefits of home-based VR training for stroke patients has not been widely researched to this point in time, but some recent studies and our data reported here do show encouraging support. For EDNA training, the pre-post effect size on the MoCA was moderate (*g* = 0.70), and triple that of the GRASP training. The training benefits of VR-based stroke intervention on cognitive function has been demonstrated in recent meta-analytic reviews, however, the vast majority of studies have evaluated clinic-based applications [8]. More targeted, cognitive-focused VR interventions in the home have shown efficacy for sub-acute stroke patients [19]. In the recent study by Torrisi and colleagues, it was notable that the cognitive training program did require that patients interact with the VR display by using pointing movements in response to memory, perception, attention and reasoning tasks. Torrisi demonstrated strong cognitive benefits (on the MoCA) following an extended 6-month training regime, the rate of change being higher than conventional training using non-VR methods; motor functions were not evaluated, however.

The combined motor and cognitive benefits of the EDNA-22 system (over and above the active control group training) are likely to facilitate the transfer of training effects to general behavioural functioning (*g* = 0.57 on the SIS, *g* = 0.49 on the NFI). Collectively, our results and that of other recent studies [19, 48, 49] give confidence that customized take-home interventions that enlist VR-based technologies can make a significant impact on the functioning of patients, augmenting more conventional practices. What is needed are larger-scale trials and a better understanding of the factors that support their implementation. We argue that transfer of home-based rehabilitation (using EDNA-22) to everyday function can be enhanced by methods that better prepare caregivers in the use of self-administered rehabilitation technologies [50]. Such technologies need to become part of the *health literacy* of all stakeholders who are impacted by stroke and other acquired brain injuries. Recent evidence supports the value of engaging and educating both patients and caregivers in all stages of rehabilitation and its methods [51]. However, we require further evidence to evaluate the precise therapeutic benefits of user engagement and health literacy when implementing home-based rehabilitation.

### Limitations

The scale of the RCT places some constraints on the inferences we can draw about the transfer of training effects and their maintenance over time. That our treatment effects were medium-to-large provide encouragement, but small samples do limit the generalizability of findings to the full spectrum of patients who suffer an ABI. In particular, the participants in the current study had mild-to-moderate neurological deficits, and we need to understand issues with implementation that may occur with patients who have more severe post-stroke disability. The impact of factors that are common in the community, such as English as a second language or patients who have more limited support from family caregivers, requires better understanding. As well, we were unable to administer a more comprehensive battery of motor and cognitive tests (esp. at follow-up testing) in lieu of restrictions on the study that were imposed by the COVID pandemic. Finally, since participants had the option of completing 3–4 sessions per week, the total number of sessions completed per week over the course of training could vary. However, we determined that there was no significant difference in total sessions between groups. Notwithstanding this, the large treatment effect that we observed on our primary motor measure (BBT) and the associated cognitive benefits (MoCA) of training does provide some confidence in the value of EDNA-22, and impetus for a larger-scale RCT and/or implementation trial.

## Conclusions

Taken together, the results of this RCT provide very encouraging support for the efficacy of the take-home EDNA-22 system for stroke rehabilitation. Results are broadly consistent with earlier evaluations of the *Elements* tabletop system, implemented in the clinic setting [13]—significant changes in motor and cognitive performance, and encouraging trends on functional behaviour. The strong predictive validity of both the BBT and MoCA suggest that the training benefits of EDNA-22 are very likely to transfer to everyday functional behaviour. These results are largely consistent with the most recent systematic reviews of home-based VR technologies for stroke [52] which suggest both cost savings of VR (relative to TAU) and comparable efficacy relative to clinic-based treatments. A larger implementation trial is planned to evaluate the active ingredients of the system, and the added benefit of e-health literacy for longer-term recovery.

## Data Availability

The datasets used and/or analyzed during the current study are available from the corresponding author on reasonable request.

## Abbreviations

ABI: Acquired brain injury
AF: Augmented feedback
BBT: Box and Blocks Test
CI: Confidence Interval
GMLT: Groton Maze Learning Task
ICF: International Classification of Functioning
MoCA: Montreal Cognitive Assessment
NFI: Neurobehavioral Functioning Inventory
NIHSS: National Institutes of Health Stroke Scale
PROM: Patient-reported outcome measure
SST: Set Shift Task
TAU: Treatment as usual
VR: Virtual reality
